# Hyperinsulinemia enhances c-Myc-mediated mammary tumor development and advances metastatic progression to the lung in a mouse model of type 2 diabetes

**DOI:** 10.1186/bcr3089

**Published:** 2012-01-07

**Authors:** Rosalyn D Ferguson, Ruslan Novosyadlyy, Yvonne Fierz, Nyosha Alikhani, Hui Sun, Shoshana Yakar, Derek LeRoith

**Affiliations:** 1 Division of Endocrinology, Diabetes and Bone Diseases, The Samuel Bronfman Department of Medicine, Mount Sinai School of Medicine, New York, NY 10029, USA

## Abstract

**Introduction:**

Hyperinsulinemia, which is common in early type 2 diabetes (T2D) as a result of the chronically insulin-resistant state, has now been identified as a specific factor which can worsen breast cancer prognosis. In breast cancer, a high rate of mortality persists due to the emergence of pulmonary metastases.

**Methods:**

Using a hyperinsulinemic mouse model (MKR^+/+^) and the metastatic, c-Myc-transformed mammary carcinoma cell line Mvt1, we investigated how high systemic insulin levels would affect the progression of orthotopically inoculated primary mammary tumors to lung metastases.

**Results:**

We found that orthotopically injected Mvt1 cells gave rise to larger mammary tumors and to a significantly higher mean number of pulmonary macrometastases in hyperinsulinemic mice over a period of six weeks (hyperinsulinemic, 19.4 ± 2.7 vs. control, 4.0 ± 1.3). When Mvt1-mediated mammary tumors were allowed to develop and metastasize for approximately two weeks and were then surgically removed, hyperinsulinemic mice demonstrated a significantly higher number of lung metastases after a four-week period (hyperinsulinemic, 25.1 ± 4.6 vs. control, 7.4 ± 0.42). Similarly, when Mvt1 cells were injected intravenously, hyperinsulinemic mice demonstrated a significantly higher metastatic burden in the lung than controls after a three-week period (hyperinsulinemic, 6.0 ± 1.63 vs. control, 1.5 ± 0.68). Analysis of Mvt1 cells both *in vitro *and *in vivo *revealed a significant up-regulation of the transcription factor c-Myc under hyperinsulinemic conditions, suggesting that hyperinsulinemia may promote c-Myc signaling in breast cancer. Furthermore, insulin-lowering therapy using the beta-adrenergic receptor agonist CL-316243 reduced metastatic burden in hyperinsulinemic mice to control levels.

**Conclusions:**

Hyperinsulinemia in a mouse model promotes breast cancer metastasis to the lung. Therapies to reduce insulin levels in hyperinsulinemic patients suffering from breast cancer could lessen the likelihood of metastatic progression.

## Introduction

Breast cancer incidence and progression are affected by several lifestyle factors, such as hormone therapy, body mass index, dietary intake and physical activity [1]. Type 2 diabetes (T2D) is an emerging major health concern, affecting around 285 million adults worldwide and predicted to affect up to 439 million by 2030 [2]. Epidemiological studies have recently demonstrated that the risks for breast cancer incidence and mortality are increased in individuals suffering from T2D [3-6]. A prolonged phase of pre-diabetes usually occurs before the onset of officially diagnosed T2D in which the main components of the metabolic syndrome, including dyslipidemia, hyperglycemia and hyperinsulinemia may be present for many years. For hyperinsulinemia, specifically, a positive correlation has recently been reported with breast cancer incidence [7,8].

An array of human breast cancer specimens have been found to harbor high expression of the insulin receptor (IR) subtype A [9-11], which is involved in the mitogenic response to insulin, as opposed to IR-B which plays a major role in metabolism [12]. Likewise, *in vitro*, numerous studies have reported that breast cancer cell lines proliferate in response to insulin [13-15].

In the last few years our laboratory has been studying a mouse model of type 2 diabetes, which manifests hyperinsulinemia and dyslipidemia, namely the MKR^+/+ ^mouse model. MKR^+/+ ^mice were generated a decade ago [16] by overexpression of a kinase dead insulin-like growth factor-1 receptor (IGF-IR) specifically in muscle under control of the creatine kinase promoter. Hyperinsulinemic MKR^+/+ ^female mice demonstrated enhanced mammary gland ductal branching and increased lateral bud formation. Growth and progression of orthotopic- and genetically-induced mammary tumors in female MKR mice were accelerated as compared to controls, but were blocked using pharmacological inhibitors of insulin signaling or insulin-sensitizers [17,18].

A high rate of mortality from breast cancer persists due to the emergence of metastases in distant organs, commonly the lungs [19]. Although studies from our laboratory and others have shown that insulin promotes primary tumor growth, studies investigating a possible connection between insulin and metastatic events in general are limited. In this study we use the hyperinsulinemic MKR^+/+ ^mouse model to study the development of mammary tumors and metastases following orthotopic injection of a highly proliferative and metastatic murine tumor cell line Mvt1, which, like many tumor types, over-expresses the transcription factor c-Myc. In MKR^+/+ ^mice, not only do Mvt1-mediated mammary tumors develop more rapidly, but the incidence of Mvt1-mediated pulmonary metastases is significantly higher. Mvt1 cells, both *in vivo *and *in vitro*, respond to hyperinsulinemia with increased expression of the transcription factor c-Myc, suggesting that high levels of insulin could increase the activity of this oncogenic factor in breast cancer. Furthermore, when we used insulin-lowering therapy in the MKR^+/+ ^mice harboring Mvt1 cells, lung metastatic burden was reduced to control levels.

## Materials and methods

### Animal studies

Mice were housed four per cage in a clean mouse facility, fed a standard mouse chow (Purina Laboratory Chow 5001; Purina Mills (**St. Louis, Missouri, USA**) and water *ad libitum*, and kept on a 12-hour light:dark cycle. Animal care and maintenance were provided through the Mount Sinai School of Medicine AAALAC Accredited Animal Facility. All procedures were approved by the Animal Care and Use Committee of the Mount Sinai School of Medicine according to the National Institutes of Health Guide Line. All mice were on Friend Virus B (National Institute of Health) (FVB/N) background. For orthotopic injections, 100,000 Mvt1 cells resuspended in sterile PBS in a volume of 100 μl were injected using a 30-gauge needle into the left inguinal mammary fat pad. Tumor volume was measured with calipers until tumors reached a specified dimension for resection (30 to 40 mm^3^) or until the time of sacrifice. Tumor volume was calculated using a three-co-ordinate system using the formula: Volume = 4/3 π (length/2 × width/2 × depth/2). For analysis of pulmonary metastases, mice were sacrificed and lungs were inflated via the trachea with 10% formalin, removed and examined for macrometastatic lesions. Lungs were embedded in paraffin, sectioned and stained using haematoxylin and eosin (H & E). Intravenous cell inoculations were performed by injecting 10,000 Mvt1 cells in a total volume of 100 μl.

### Cell culture

The murine mammary cell line Mvt1 was derived from an explant culture of an MMTV c-Myc/Vegf transgenic female mouse as described elsewhere [20]. Cells were maintained in Dulbecco's Modified Eagles Medium (DMEM) supplemented with 10% Fetal Bovine Serum (FBS) (Invitrogen, Grand Island, NY, USA), 100 U/ml penicillin and 100 μg/ml streptomycin (Mediatech, Manassas, VA, USA) and grown at 37°C in 5% CO_2 _atmosphere with 95% humidity.

### Western blotting

Mvt1 cells or tumor tissues were lysed in chilled lysis buffer (pH 7.4) containing 50 mM Tris, 150 mM NaCl, 1 mM EDTA, 1.25% CHAPS, 1 mM sodium orthovanadate, 10 mM sodium pyrophosphate, 8 mM B-glycerophosphate and Complete Protease Inhibitor Cocktail tablet. Protein concentration of samples was measured using the BCA protein assay kit (Thermo Scientific, Rockford, IL, USA). Protein samples were resuspended in 3× loading buffer containing DTT (Cell Signaling Technologies, Danvers, MA, USA) and denatured by boiling for five minutes at 96°C. Samples were then subjected to SDS polyacrylamide gel electrophoresis (SDS-PAGE) and transferred to a nitrocellulose membrane. Membranes were probed with the appropriate primary antibodies: anti-phospho Akt ^(Ser473)^, Akt, c-Myc, matrix metalloprotease (MMP) -9 and β-actin (obtained from Cell Signaling Technology, Danvers, MA, USA), anti-insulin receptor (IR)-β, IGF-IR and vascular endothelial growth factor (VEGF) (obtained from Santa Cruz Biotechnology, Santa Cruz, CA, USA) before being incubated with secondary antibodies (LI-COR Biosciences, Lincoln, NE, USA) and being exposed to the LI-COR infrared detection system (LI-COR Biosciences).

#### Semi-quantitative polymerase chain reaction (PCR)

RNA was extracted from tumor tissues using the RNeasy lipid extraction kit (QIAGEN, Valencia, CA, USA) according to the manufacturer's instructions. RNA integrity was verified using a Bioanalyzer (Agilent Technologies 2100 Bioanalyzer-Bio Sizing, Version A.02.12 SI292), (Agilent Technologies, Santa Clara, CA, USA). One μg of RNA was reverse-transcribed to cDNA using oligo (dT) primers with a RT-PCR kit according to the manufacturer's instructions (Invitrogen). After reverse transcription of RNA, cDNA was subjected to PCR cycling conditions as follows: initial denaturation at 95°C for 2 minutes, 30 cycles of amplification consisting of a 15 s denaturation step at 95°C, a 30 s annealing step at 58°C, and a 1 minute extension step at 72°C. A final seven-minute extension was performed at 72°C. Primer sequences used were as follows: IGF-I 5' GGACCAGAGACCCTTTGCGGGG, IGF-I 3' GGCTGCTTTTGTAGGCT TCAGTGG, IGF-II 5' CCTTCGCCTTGTGCTGCAT, IGF-II 3' ACGGTTGGCACGGCTTAA, β-actin 5' CCTAAGGCCAACCGTGAAAA, β-actin 3' GAGGCATACAGGGACAGCACA.

### Proliferation assays

Mvt1 cells were seeded in 24-well plates at a density of 1 × 10^4 ^cells/ml and allowed to adhere for 24 hours. Standard growth medium was then exchanged for serum-free DMEM containing 0.1% BSA and cells were allowed to rest for one hour before the addition of insulin. Cells were incubated with insulin at concentrations of 10 nM or 100 nM for 72 hours and medium was changed daily. Cells were then trypsinized, diluted in trypan blue (1:2) and counted by haemocytometer using trypan blue exclusion.

### Statistical analysis

Results are expressed as means ± SEM. Statistical analyses were conducted using the Student's *t*-test and, where appropriate, two-way ANOVA followed by Tukey HSD *post-hoc *test, with *P *≤ 0.05 considered significant.

## Results

### In MKR^+/+ ^mice, orthotopically injected Mvt1 cells form larger primary tumors and exhibit more pulmonary metastases than in control mice

**Figure 1:**
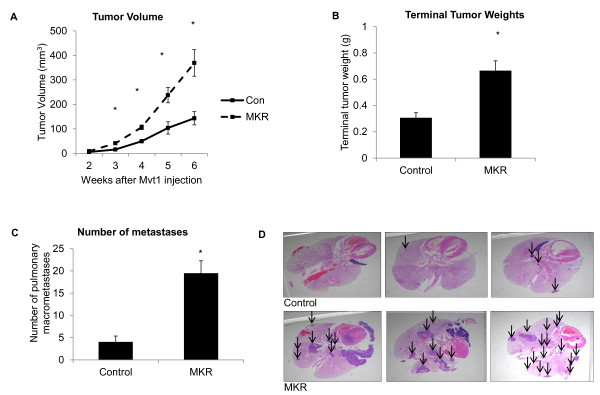
**Mvt1 tumor growth and metastasis development are more rapid in MKR^+/+ ^mice compared to controls**. A total 100,000 Mvt1 cells were injected into the left inguinal mammary fat pad of control and MKR^+/+ ^mice (8 to 10 per group). (A) Mammary tumor volume (mm^3^) measured by calipers. (B) Terminal weights of Mvt1-induced mammary tumors. (C) Numbers of pulmonary macrometastases in control and MKR^+/+ ^mice. (D) H & E staining of control and MKR^+/+ ^lungs, arrows indicate metastases. Error bars represent SEM *, *P *≤ 0.05.

The metabolic characteristics of female MKR^+/+ ^mice have been described and illustrated previously [17,18]. The principal abnormalities in these mice include severe insulin resistance and hyperinsulinemia on a background of mild dysglycemia (approximately 20% elevated blood glucose compared to controls). Female MKR^+/+^mice are non-obese and weigh moderately less than control mice. In addition, total body fat in MKR^+/+ ^is lower than in controls and inflammatory factors commonly elevated in obesity are expressed at normal levels. We injected 100,000 Mvt1 cells into the left inguinal mammary pad of 8- to-10-week-old control and MKR^+/+ ^mice and monitored growth of mammary tumors weekly. A significant increase in Mvt1-mediated mammary tumor growth was observed in MKR^+/+ ^mice compared to controls (Figure 1A), which was confirmed by the terminal weights of tumors (Figure 1B). At the time of sacrifice (six weeks after cell injection), lungs from control and MKR^+/+ ^mice were removed and macrometastases were counted. The mean number of macrometastases/mouse was significantly higher in MKR^+/+ ^mice (mean number of macrometastases = 19.4 ± 2.7) compared to controls (mean number of metastases = 4.0 ± 1.3) (Figure 1C). Lungs of representative groups of MKR^+/+ ^mice (n = 3) and control mice (n = 3) were paraffin-embedded, sectioned and stained with H & E to verify the increased incidence of metastatic lesions in MKR^+/+ ^compared to control mice (Figure 1D).

### The incidence of pulmonary metastases remains higher in MKR^+/+ ^mice compared to controls, independent of mammary tumor size

**Figure 2:**
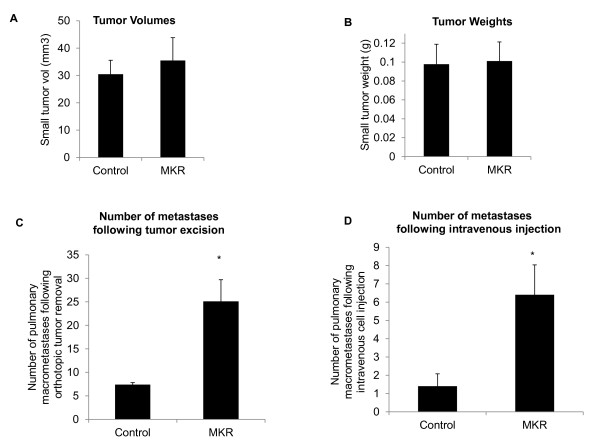
**Independent of primary tumor size, metastases formation is accelerated in MKR^+/+ ^mice**. A total of 100,000 Mvt1 cells were injected into the inguinal mammary fat pad of 8- to 10-week-old mice (8 to 10 mice per group). Developing tumors were removed surgically when they reached a specific size (30 to 40 mm^3^). (A) Volumes and (B) weights of tumors from MKR^+/+ ^and control mice at time of surgical removal. (C) Number of metastases present in the lungs of control and MKR^+/+ ^mice four weeks after tumor resection. (D) Number of metastases present in the lungs of control and MKR^+/+ ^mice three weeks after intravenous injection of 10,000 Mvt1 cells. Error bars represent SEM, **, *P *≤ 0.05.

It is possible that the significantly increased number of macrometastases in MKR^+/+ ^mice is simply a consequence of larger tumors at the primary site, which could promote greater tumor cell dissemination. To eliminate the effect of primary tumor size on metastasis occurrence, we controlled for primary tumor size. Separate cohorts of 8- to 10-week-old control and MKR^+/+ ^mice were injected in the inguinal mammary fat pad with 100,000 Mvt1 cells. Mammary tumor volume was measured with calipers twice-weekly. When tumors reached a volume of 30 to 40 mm^3 ^(14 to 16 days following cell injection, (data not shown) they were surgically removed. Tumor volumes (Figure 2A) and weights (Figure 2B) at the time of resection demonstrated no significant difference between MKR^+/+ ^and control mice.

After tumor resection, mice were returned to their original housing for a period of four weeks and were then sacrificed. Lungs from all mice were removed and pulmonary macrometastases quantified. A significant increase in the number of metastases was observed in MKR^+/+ ^mice (mean number of metastases = 25.1 ± 4.6) compared to controls (mean number of metastases = 7.4 ± 0.42) (Figure 2C).

### When equal numbers of Mvt1 cells are injected intravenously into control and MKR^+/+ ^mice, lung metastatic burden is higher in MKR^+/+ ^mice

In MKR^+/+ ^mice, insulin levels are increased and may promote mitogenesis through activation of the IR. To address whether metastatic lesions in the lungs of MKR^+/+ ^mice exhibit greater proliferative activity than those in the lungs of control mice, an equal amount of Mvt1 cells (10,000) were injected intravenously into both MKR^+/+ ^and control mice. After three weeks, all mice were sacrificed and examined for pulmonary metastases. Significantly more metastases were observed in lungs of MKR^+/+ ^mice compared to controls (MKR^+/+^, 6 ± 1.63 vs. control, 1.5 ± 0.68) (Figure 2D) indicating that the environment of the hyperinsulinemic lung is more permissive for the proliferation of circulating tumor cells.

### Mammary tumors express elevated levels of c-Myc, MMP-9, IR, IGF-IR and VEGF

**Figure 3:**
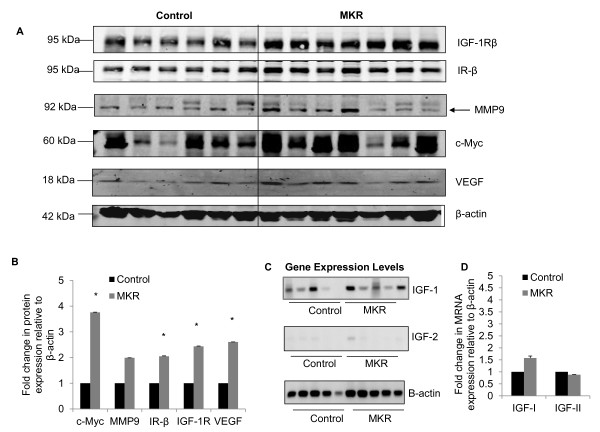
**Hyperinsulinemia results in increased expression of c-Myc, MMP-9, IR-β, IGF-IR and VEGF in Mvt1 tumors**. (A) Tumor tissue was extracted from control and MKR^+/+ ^mice and subjected to SDS PAGE and Western blotting with antibodies to c-Myc, MMP-9, IR-β, IGF-IR and VEGF. β-actin was included as a loading control. (B) Fold change in expression of c-Myc, MMP-9, IR-β, IGF-IR and VEGF in control and MKR^+/+ ^tumor tissue compared to β-actin control. *, *P *≤ 0.05. (C) Semi-quantitative PCR showing relative mRNA expression levels of *Igf1, Igf-2 *and β-actin in tumor tissue from control and MKR^+/+ ^mice. (D) Densitometric quantification of DNA gel electrophoresis showing mRNA expression of *Igf1 *and *Igf2 *relative to β-actin.

In order to examine the effects of hyperinsulinemia on the metastasis of tumor cells from the primary site, we examined levels of c-Myc, MMP-9, IR, IGF-IR and VEGF in tumor tissue extracted from control and MKR^+/+ ^mice. As shown in Figure 3A, B, in MKR^+/+ ^mice, expression levels of c-Myc, IR, IGF-IR and VEGF were significantly up-regulated compared to controls. MMP-9 expression was also elevated, although overall, a statistically significant increase was not observed. As hyperinsulinemia can also affect the expression level of IGFI and IGFII, we used semi-quantitative PCR to compare the levels of both *Igf1 *and *Igf2 *mRNA in MKR^+/+ ^and control mice. As shown in Figure 3C, D, we did not detect any difference in expression of *Igf1 *in tumors from MKR^+/+ ^and control mice. We found *Igf*2 levels to be barely detectable in tumors and could thus not observe any significant difference in levels of *Igf2 *mRNA in MKR^+/+ ^versus control mice.

### Insulin stimulates Akt phosphorylation and increased proliferation in mammary tumor cell line Mvt1 *in vitro*, and increases the expression of c-Myc and MMP-9

**Figure 4:**
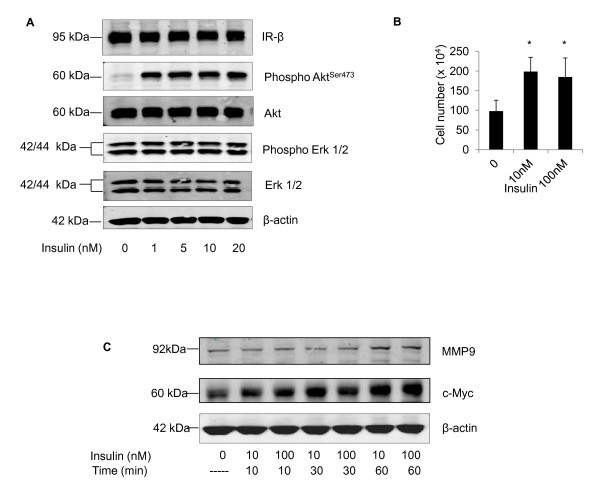
**In Mvt1 cells, insulin elevates c-Myc, MMP-9 and phosphorylated Akt, and results in increased proliferation**. (A) Akt phosphorylation in Mvt1 cells after insulin stimulation. Mvt1 cells were serum-starved for four hours prior to stimulation with increasing concentrations of insulin. Cells were then harvested and subjected to SDS PAGE and Western blotting with antibodies to insulin receptor-β (IR-β), phosphor-Akt^Ser473 ^and Akt, phospho-p42/p44^Thr202Tyr/204 ^and p42/p44. Β-actin was included as a loading control. (B) Mvt1 cells were seeded at a density of 1 × 10^4 ^cells/ml in 24-well plates and stimulated with either 10 nM or 100 nM insulin in growth medium containing 0.5% FBS. After 72 hours, cells were trypsinized and counted using a haemocytometer and trypan blue exclusion. Cell counts are representative of three independent experiments. Error bars represent SEM, *, *P *≤ 0.05. (C) Mvt1 cells were stimulated with 10 nM or 100 nM insulin for between 10 and 60 minutes. Previously, cells were grown in medium containing 0.5% FBS for 72 hours. After stimulation, cells were harvested and subjected to SDS PAGE and Western blotting with antibodies to c-Myc and MMP-9. β-actin was included as a loading control.

In order to find a potential mechanism to explain why Mvt1 cells grow faster and metastasize more readily in the presence of elevated insulin, we studied their reaction to insulin stimulation *in vitro*. Initially we verified that Mvt1 cells demonstrated a typical response to insulin stimulation. Short-term stimulation of Mvt1 cells with up to 20 nM insulin caused robust phosphorylation of Akt (Figure 4A), a central signaling protein and key mediator of insulin action downstream of the IR, demonstrating that Mvt1 cells respond appropriately to insulin. We observed no change in the phosphorylation of Erk1 and Erk2 in response to insulin stimulation. To determine whether insulin also affects the proliferation of Mvt1 cells *in vitro*, cells were seeded at a density of 1 × 10^4 ^cells/ml in 24-well plates and stimulated with 10 nM or 100 nM insulin for 72 hours. Viable cell number was assessed by trypan blue exclusion using a haemocytometer. The presence of insulin at both 10 nM and 100 nM was sufficient to cause a significant increase in the proliferation of Mvt1 cells *in vitro *(Figure 4B).

Mvt1 cells, like many other tumor types, over-express c-Myc, a transcription factor which is regulated by several extracellular growth factors [21]. We investigated whether insulin could alter the expression of c-Myc in Mvt1 cells *in vitro*. Cells were serum-starved for 72 h and then stimulated with 10 nM and 100 nM insulin for up to one hour. As shown in Figure 4C, exposure of Mvt1 cells to insulin caused an increase in the expression levels of c-Myc. Mvt1 cells are a highly metastatic cell line; we thus also investigated whether MMP-9, a key protein involved in cell metastasis from primary tumors, was affected by insulin stimulation *in vitro*. As shown in Figure 4C, insulin stimulation increased MMP-9 levels, demonstrating that insulin may also enhance cell signaling pathways which lead to metastasis from the site of primary tumors in breast cancer.

### Insulin-reducing therapy decreases the incidence of pulmonary metastases in MKR^+/+ ^mice

**Figure 5:**
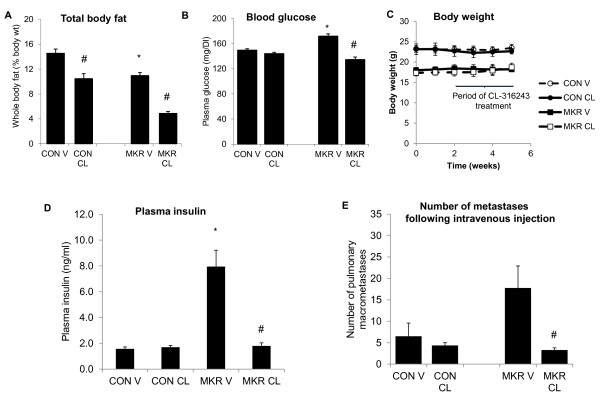
**Chronic CL-316243 treatment reduces the hyperinsulinemia and metastases burden in MKR^+/+ ^mice**. The authors treated 8- to 10-week-old MKR^+/+ ^and control mice with CL-316243 or vehicle for three weeks (6 to 8 mice per group). (A) Whole body fat (% of total body weight) was measured after 10 days of treatment. (B) Blood glucose was measured after seven days of treatment. (C) Body weight was measured weekly. (D) Serum insulin was measured after 21 days of treatment. Data are expressed as SEM. *, *P *≤ 0.05 and for control vs. MKR. ^#^, *P *≤ 0.05 for CL-316243 vs. vehicle. *(E) *Mvt1 cells (10,000) were injected intravenously into 8- to 10-week-old control and MKR^+/+ ^mice (7 to 9 per group). Mice were then treated with CL-316243 or vehicle for three weeks. Mice were sacrificed and the numbers of lung macrometasatases were counted. Data are expressed as SEM. *, *P *≤ 0.05 for control vs. MKR^+/+^. ^#^, *P *≤ 0.05 for CL-316243 vs. vehicle.

To examine whether the increased metastatic burden in MKR^+/+ ^mice was insulin-mediated, we used a pharmacological agent to lower insulin levels and then observed the result of this intervention on metastases formation in the lung. CL-316243 is a potent and highly selective β_3_-adrenoceptor agonist, which decreases blood insulin and glucose levels following oral administration *in vivo *[22]. In our previous studies we have shown that CL-316243 dramatically improved the diabetic state in male MKR^+/+ ^mice by reducing circulating glucose and insulin levels and enhancing whole body metabolic rate [23]. Furthermore, we have shown that CL-316243 treatment in female MKR^+/+ ^mice, caused a significant decrease in the growth rate of both PyVmT and Neu/ErbB2 cell-mediated mammary tumors [17]. To assess the effect of CL-316243 treatment on the metastatic growth of Mvt1 cells, both MKR^+/+ ^and control mice were injected intravenously with 10,000 Mvt1 cells. Mice were then treated with either CL-316243 (1 mg·kg body wt^-1 ^day^-1^) or vehicle for three weeks. During the CL-316243 or vehicle treatment period, we monitored metabolic parameters. As shown in Figure 5A, MKR^+/+ ^mice demonstrated a significant reduction of total fat mass as well as a reduction in serum insulin levels (Figure 5A, D) and a concomitant decrease in blood sugar (Figure 5B). In control mice, no change in insulin levels was observed (Figure 5D). Throughout the CL-316243 treatment, no change in body weight was observed in control or MKR^+/+ ^mice (Figure 5C), consistent with our previous observations [17].

At the end of the treatment period, all mice were sacrificed and numbers of macrometastases were quantified. As shown in Figure 5E, we observed a significant reduction in the numbers of Mvt1-mediated metastases in MKR^+/+ ^mice treated with CL-316243 and no effect in controls, suggesting that reductions in insulin levels, independent of primary mammary tumor size, could directly lower the incidence of Mvt1 metastases in MKR^+/+ ^mice.

## Discussion

Several epidemiological studies have demonstrated that the risks of breast cancer incidence and mortality are both positively associated with type 2 diabetes (T2D), a multi-factorial disease encompassing several metabolic dysfunctions, such as insulin resistance and hyperinsulinemia, hyperglycemia and dyslipidemia [3-6,24]. Additionally, hyperinsulinemia, specifically, is a significant risk factor for breast cancer incidence, independent of other factors associated with type 2 diabetes [7]. Breast cancer mortality rates remain high, primarily due to the metastasis of primary tumors to distant organs, such as the lungs [25]. In type 2 diabetic patients, it is possible that breast cancer metastasis may also be augmented by metabolic dysfunctions; thus we investigated in a mouse model whether hyperinsulinemia, specifically, affects the metastasis of primary mammary tumors to the lung.

We used the female MKR^+/+ ^mouse, which manifests severe insulin resistance and hyperinsulinemia, yet is only mildly hyperglycemic and leaner than controls. Previous work from our laboratory has established that two different murine mammary tumor cell lines (Met1 and MCNeuA) develop significantly larger orthotopic tumors in MKR^+/+ ^mice compared to controls, demonstrating a potent effect of hyperinsulinemia on mammary tumor development [17,18]. We now use an alternative mouse mammary tumor cell line, Mvt1, which spontaneously metastasizes from orthotopically-induced mammary tumors in order to study the effect of hyperinsulinemia, during type 2 diabetes, on the progression of primary tumors to metastases. When inoculated, Mvt1 cells form significantly larger tumors in MKR^+/+ ^mice than in controls, a finding which reinforces our previous data on hyperinsulinemia and mammary tumor development. Furthermore, the number of spontaneous metastases in the lungs of MKR^+/+ ^mice is also greater than in controls, demonstrating a positive association between hyperinsulinemia and mammary tumor metastasis.

A relationship between hyperinsulinemia and breast cancer progression to metastasis has not been verified by clinical studies. However, experimental data indicate that the major events of primary tumor metastasis, such as migration, invasion and angiogenesis, are enhanced by elevated insulin levels. Chinese Hamster Ovary (CHO) cells overexpressing the IR become highly chemotactic toward insulin stimulation [26]**. **Insulin increases the migration and invasion of human hepatocarcinoma cell line H7721, and its adhesion to human umbilical vein endothelial cells (HUVEC). Furthermore, these metastases-related effects can be reversed by the addition of an inhibitor to phosphatidylinositide 3-kinase (PI3-K), one of the main signaling molecules downstream of activated IR [27]. In an *in vivo *study of orthotopically-induced mouse mammary tumors progressing to lung metastases, down-regulation of the IR in tumor cells results in reduced primary tumor growth and fewer pulmonary lesions, along with diminished angiogenesis, demonstrating an important role for insulin signaling in cancer progression [28].

We observed more spontaneous metastases in the lungs of MKR^+/+ ^mice compared to controls; however, we identified that this finding could be due simply to the larger tumors in MKR^+/+ ^mice, rather than a response to insulin. Our surgical removal of tumors from MKR^+/+ ^and control mice when they reached a specific size (35 to 40 mm^3^) demonstrated that more metastasis had already occurred in MKR^+/+ ^mice compared to controls, apparent from the greater number of metastases which were later detected in the lungs. This demonstrates the potent effect of elevated insulin in advancing the metastatic spread of tumor cells.

Our analysis of tumor tissue reveals increased c-Myc expression in tumors of MKR^+/+ ^mice compared to controls. c-Myc is a transcription factor which controls cell-cycle progression, metabolism and differentiation, and is expressed at low levels in normal resting cells [29]. Activation of c-Myc depends on its formation of a heterodimeric complex with Max [30]. Around 50% of breast cancers are a consequence of c-Myc-driven oncogenic transformation, which occurs by gene amplification, chromosomal translocation or protein overexpression and stabilization [29,31-33]. Certain growth factors augment oncogenic c-Myc expression in human breast cancer, including TGFα and IGF-I [34-36]. Insulin can up-regulate c-Myc in the estrogen-driven human breast cancer cell line MCF-7, which is augmented by the addition of estradiol [37]. Additionally, both insulin and IGF-I have been reported to stimulate expression of c-Myc in non-transformed bovine fibroblast cells in culture [38]. To our knowledge, there are no reports of insulin influencing c-Myc levels in an *in vivo *setting; thus, we believe that ours are the first data to demonstrate this association. In our model, we observed elevated c-Myc expression in the presence of high insulin levels both *in vivo *and *in vitro*. We also observed increased tumor mass and cell proliferation, respectively, suggesting a role for insulin in significantly promoting the growth-mediating effects of c-Myc.

c-Myc is integrally involved in breast cancer metastasis, promoting loss of apoptosis, invasion, and angiogenesis [39-42]. Thus, in addition to accelerating cell proliferation, insulin-mediated increases in c-Myc expression could potentially enhance metastatic events. Indeed, in our model, increased c-Myc was associated with elevated levels of MMP-9 and VEGF, which are both important mediators of metastatic events *in vivo *[43,44]. MMP-9 is a key protease secreted from metastatic cells, which implements proteolytic modification or degradation of the extracellular matrix during tumor cell dissemination [45]. Knock-down of c-Myc in a murine lung cancer model led to a reduction in MMP-9 levels and diminished metastasis of lung tumor cells to distant sites [46]. It has also been reported that MMP-9 is a direct target of c-Myc in cultured murine lymphoid endothelial cells during the initiation and progression of atherosclerotic lesions [47].

VEGF is a key mediator of angiogenesis and is essential for intravasation of metastasizing tumor cells [43] as well as for primary tumor development [48]. Transgenic mice overexpressing c-Myc in the mammary gland resulted in low rates of lung micrometastases, whereas when c-Myc and VEGF were expressed simultaneously, high rates of macrometastases occurred [49]. In the ascites of patients with metastatic ovarian cancer, lysophosphatidic acid (LPA) stimulated expression of VEGF, an event which was completely dependent on c-Myc expression [50]. In agreement with these data, we observed increased VEGF in tumor tissue which also expressed elevated levels of c-Myc.

We also demonstrated a significant up-regulation of the IR and an elevation of the IGF-IR in tumors from MKR^+/+ ^mice. Although human clinical studies have not investigated levels of the IR in breast tumors from hyperinsulinemic patients, specifically, it has been reported that IR expression, as well as being a strong predictor of poor survival rate, spans all three subsets of clinical breast cancer (luminal, Her2 positive and triple negative) [9,10], and mammary tumorigenesis in mice resulting from transgenic expression of *Neu, Wnt1*, or *Ret *oncogenes is accompanied by significant elevations of IR levels in all three tumor types [51]. These data all suggest that increased IR expression is linked to the onset or development of breast cancer. The IGF-IR is highly homologous to the IR, activates similar signaling pathways when bound by IGFI/II, and has a well-established role in the progression of breast cancer [52]. It has been reported that insulin itself can increase IGF-IR levels [53]. Hyperinsulinemia is also known to increase circulating IGF1 production, either by up-regulating growth hormone receptor levels [54] or by suppressing IGF binding protein (IGFBP) -1 and -2 [55]. However, we found no significant difference in *Igf1 *mRNA levels in the tumor tissue of MKR^+/+ ^and control mice, suggesting that any differences in tumor growth or metastases formation were due to insulin rather than IGF-I. It is known that IGF-II causes activation of the IR [12]; but its expression is confined mainly to fetal development and thus circulates at extremely low levels postnatally in rodents [56]. Indeed, in tumor tissue from both control and MKR^+/+ ^mice we observed barely detectable levels of *Igf2 *mRNA expression.

Our finding of increased metastatic burden following intravenous injection of Mvt1 cells in the absence of a primary tumor suggests that hyperinsulinemia promotes increased survival or proliferation (or both) of circulating tumor cells that arrest in the lungs. Our *in vitro *data confirm that insulin stimulates the expected canonical Akt signaling pathway in Mvt1 cells and also enhances Mvt1 proliferation. The significantly reduced number of lung metastases, which we observed after three weeks of insulin-lowering treatment with CL-316243, suggests that reducing insulin levels causes a decrease in either survival or proliferation of Mvt1 cells in the lungs. In agreement with this finding, previous work from our laboratory has reported an abrogation of both PyVmT- and Neu- mediated orthotopic mammary tumor growth during chronic CL-316243 treatment [17].

In summary, we have used a hyperinsulinemic mouse model to study the effect of elevated systemic insulin levels on mammary tumor metastasis to lung. As well as confirming previously published data whereby insulin accelerates primary tumor growth, we also provide important new findings which suggest that insulin also affects breast cancer progression to the metastatic stage. This indicates that breast cancer patients presenting with hyperinsulinemia may be at increased risk of primary tumor progression to lung metastases. These data could explain, in part, the increased mortality in patients with breast cancer and T2D and highlights the benefit of using insulin-reducing therapies to reduce the mortality risk from the combined effect of these two diseases.

## Conclusions

Hyperinsulinemia *in vivo *can significantly increase both primary tumor growth and subsequent metastasis to the lung in a mouse model. During periods of hyperinsulinemia, the lungs may provide an environment which can augment the survival or proliferation of metastatic cells. Oncogenic c-Myc expression is increased during periods of hyperinsulinemia, both *in vivo *and *in vitro *and this could contribute both to primary tumor growth and metastatic events. Finally, insulin-reducing treatment can significantly diminish the growth of intravenously inoculated metastatic cells in the lungs, suggesting that therapies to lower insulin in breast cancer patients presenting with hyperinsulinemia could be a valuable strategy to reduce mortality from breast cancer metastatic progression.

## Abbreviations

CHAPS: 3-((3-cholamidopropyl)dimethylammonio)-1-propanesulfonate; CHO: Chinese Hamster Ovary; DTT: dithiothreitol; EDTA: ethylene diamine tetra-acetic acid; HUVEC: human umbilical vein endothelial cells; IGF-IR: insulin-like growth factor 1 receptor; IR: insulin receptor; LPA: lysophosphatidic acid; MKR: (MCK) muscle creatinine kinase: KR (arg to lys substitution to create a dominant negative IGF-IR); MMP: matrix metalloprotease; PI3-K: phosphatidylinositide 3-kinase; PyVmT: polyoma virus middle T antigen; T2D: type 2 diabetes; VEGF: vascular endothelial growth factor

## Competing interests

The authors declare that they have no competing interests.

## Authors' contributions

RF performed *in vivo *and *in vitro *experiments, experimental design and strategies, and manuscript preparation. RN obtained Mvt1 cells, contributed to experimental design and strategies, provided preliminary data on metastatic potential of Mvt1 cells, and contributed to/edited the manuscript. YF contributed to experimental strategies, provided training on animal injections/surgery, and contributed to/edited the manuscript. NA provided assistance with *in vivo *procedures/animal surgery, performed RNA extraction and PCR reactions, and contributed to editing of the manuscript. HS provided training in animal surgery/techniques. SY contributed to experimental strategies, training on animal surgery/techniques, and contributed to/edited the manuscript. DL is the Principal Investigator and corresponding author on the project, obtained funding, and contributed to experimental strategies and editing of/contributions to the manuscript. All authors read and approved the final manuscript.
